# The glucose transporter GLUT12, a new actor in obesity and cancer

**DOI:** 10.1007/s13105-024-01028-9

**Published:** 2024-05-10

**Authors:** Miguel Burgos, Eva Gil-Iturbe, Adrián Idoate-Bayón, Rosa Castilla-Madrigal, Maria J. Moreno-Aliaga, M. Pilar Lostao

**Affiliations:** 1 Center for Nutrition Research and Department of Nutrition, Food Science and Physiology; School of Pharmacy and Nutrition, University of Navarra, Pamplona, Spain; 2 IdiSNA, Navarra Institute for Health Research, Pamplona, Spain; 3 CIBER de Fisiopatología de la Obesidad y Nutrición (CIBEROBN), Instituto de Salud Carlos III (ISCIII), Madrid, Spain; 4 Present Address: Department of Psychiatry, Columbia University Irving Medical Center, New York, NY USA

**Keywords:** GLUT12, Obesity, Cancer, Warburg effect

## Abstract

Obesity constitutes a global health epidemic which worsens the main leading death causes such as type 2 diabetes, cardiovascular diseases, and cancer. Changes in the metabolism in patients with obesity frequently lead to insulin resistance, along with hyperglycemia, dyslipidemia and low-grade inflammation, favoring a more aggressive tumor microenvironment. One of the hallmarks of cancer is the reprogramming of the energy metabolism, in which tumor cells change oxidative phosphorylation to aerobic glycolysis or “Warburg effect”. Aerobic glycolysis is faster than oxidative phosphorylation, but less efficient in terms of ATP production. To obtain sufficient ATP, tumor cells increase glucose uptake by the glucose transporters of the GLUT/SLC2 family. The human glucose transporter GLUT12 was isolated from the breast cancer cell line MCF7. It is expressed in adipose tissue, skeletal muscle and small intestine, where insulin promotes its translocation to the plasma membrane. Moreover, GLUT12 over‐expression in mice increases the whole‐body insulin sensitivity. Thus, GLUT12 has been proposed as a second insulin‐responsive glucose transporter. In obesity, GLUT12 is downregulated and does not respond to insulin. In contrast, GLUT12 is overexpressed in human solid tumors such as breast, prostate, gastric, liver and colon. High glucose concentration, insulin, and hypoxia upregulate GLUT12 both in adipocytes and tumor cells. Inhibition of GLUT12 mediated Warburg effect suppresses proliferation, migration, and invasion of cancer cells and xenografted tumors. This review summarizes the up-to-date information about GLUT12 physiological role and its implication in obesity and cancer, opening new perspectives to consider this transporter as a therapeutic target.

## Introduction

Obesity, which has multiplied its incidence in the last decades, constitutes a global health epidemic. In obesity, an excessive accumulation of dysfunctional adipose tissue leads to the development of many comorbidities, such as type 2 diabetes mellitus, non-alcoholic fatty liver disease and cardiovascular disorders, among others [20]. Remarkably, obesity also increases the risk to suffer different types of cancers, including breast, liver, colorectal and ovarian [25].

The pathophysiology of obesity frequently leads to insulin resistance, hyperinsulinemia, hyperglycemia, dyslipidemia and low-grade chronic inflammation. These factors favor an aggressive tumor microenvironment. Indeed, the secretion of proinflammatory cytokines, adipokines and estrogens by dysfunctional adipocytes contributes to initiation, progression and recurrence of tumors in subjects with obesity [50, 52]. The deregulation of molecules within the microenvironment milieu increases the tumor activity through JAK-STAT, MAPK and PI3K signaling pathways, the main actors for growth, proliferation and apoptosis evasion in tumor cells [32, 38].

Reprogramming the energy metabolism is recognized as a hallmark of cancer [28]. Among other changes, tumor cells drive their catabolic glycolytic signaling pathway to produce lactate in the presence of oxygen, a process known as aerobic glycolysis or “Warburg effect” [71]. Aerobic glycolysis is faster than oxidative phosphorylation, but less efficient in terms of ATP production. Therefore, to produce sufficient ATP via glycolysis, tumor cells need to increase glucose uptake and metabolism. Glucose uptake in tumor cells is carried out by facilitative transport across the plasma membrane mediated by the sugar transporters of the GLUT/SLC2 family [5]. Of note, the uptake through these transporters has been established as the rate-limiting step for ATP production through glucose metabolism [30].

### The GLUT family of glucose transporters

The GLUT/SLC2 (SoLute Carrier) protein family of facilitative glucose transporters mediates the bidirectional transport of monosaccharides across the plasma membrane down their concentration gradient, being the main actors in the maintenance of glucose homeostasis within the body. Fourteen different members of the SLC2 family have been identified. They are grouped into three classes based on their primary sequence homology: class I includes GLUT1-4 and GLUT14; class II comprises GLUT5, 7, 9 and 11; and class III includes GLUT6, 8, 10, 12, and the H^+^/myo-inositol cotransporter HMIT. The subcellular location, level of expression, and regulation of each GLUT is specific for each tissue according to the metabolic needs of the cells, and to allow the appropriate distribution of whole-body glucose [3, 31]. Mutations and/or dysregulation of GLUT proteins are the cause or are associated with a variety of diseases [2, 3, 31, 53].

The isoforms of class I were the first cloned and characterized, and their physiological roles, together with that for GLUT5, are well established. However, the function in the organism of the rest of GLUTs still is under investigation. GLUT1 is expressed in red blood cells, blood–brain barrier, brain, kidney, placenta, and in the rest of the organism at different levels, being its main function the maintenance of basal glucose concentration. GLUT2 can also transport galactose and fructose; it is expressed in the liver, pancreatic beta cells, and basolateral membrane of the renal and intestinal epithelial cells for glucose re/absorption. In the pancreas, its function is to transport glucose during the postprandial periods to stimulate the release of insulin. GLUT3, together with GLUT1, is the main glucose transporter in the brain. GLUT4 is located intracellularly in insulin-sensitive tissues, and translocates to the membrane in response to insulin to transport glucose into the cell. GLUT5 is the main fructose transporter; it is expressed in the brush border of the intestinal epithelium but also in red blood cells, adipose tissue, skeletal muscle, spermatozoa and kidney [3, 31].

### The glucose transporter GLUT12

The human glucose transporter GLUT12 (SLC2A12), one of the latest GLUT transporters identified, belongs to the class III of the facilitative glucose transporter family SLC2 [42]. It was isolated from the breast cancer cell line MCF7 by its homology with GLUT4, the insulin sensitive glucose transporter [59].

Using radiolabeled-sugars uptake and electrophysiological methods applied to GLUT12-expressing *Xenopus laevis* oocytes, studies from our laboratory demonstrated that glucose transport increases by 50% in the presence of Na^+^. Furthermore, glucose induces chloride currents that are uncoupled to glucose transport. GLUT12 shows low sugar selectivity transporting: D-glucose > α-methyl-D-glucose (αMG) > 2-deoxy-D-glucose (2-DOG) > D-galactose > D-fructose [54]; being αMG the classical substrate of the Na^+^/glucose cotransporters SGLTs, not transported by any of the other GLUT transporters [9]. Studies performed in GLUT12-reconstituted proteo-liposomes show a glucose K_m_ of 6.4 mM, which is half of that for GLUT1 [45].

As GLUT4 (class I) and the rest of the members of class III, GLUT12 contains a dileucine motif in the N and C-terminal domains, that retains the transporter in intracellular compartments, mostly in perinuclear regions associated with the Golgi network, in the absence of insulin stimulus [1, 59]. In line with these data, GLUT12 protein is expressed in white adipose tissue, skeletal muscle, and small intestine, all insulin-responsive tissues, where insulin promotes its translocation to the plasma membrane [21, 22, 67]. Accordingly, the promoter region of hGLUT12 contains insulin response elements [22]. In addition, GLUT12 over‐expression in mice increases the whole‐body insulin sensitivity and glucose clearance rate in insulin‐sensitive tissues [57]. However, in Zebrafish, the lack of GLUT12 is related to heart failure and diabetic phenotype during embryonic development, in which GLUT4 is expressed later than GLUT12 [35]. Altogether, these data support the role of GLUT12 as a second insulin‐responsive glucose transporter [57, 67].

We have reported that extracellular glucose and insulin also induce rapid translocation of GLUT12 to the brush border membrane in the human intestinal epithelial cell line Caco-2 and in mice jejunum, most probably in relation with its participation in sugar absorption during postprandial periods. In these cells, GLUT12 trafficking to the apical membrane and sugar uptake are increased by activation of AMPK [22], a cellular energy sensor that plays a key role in energy cell homeostasis [29]. Interestingly, it has been reported the GLUT12 unresponsiveness to insulin in the heart of diabetic mice, where GLUT4 would be the only GLUT transporter sensitive to insulin [73]. However, the authors found the highest expression of GLUT12 in the cell membrane, possibly trying to compensate for the lack of GLUT4 due to insulin resistance [73].

GLUT12 is also expressed in the cytoplasm and apical membrane of distal tubules and collecting ducts of human and rat kidney [39]. This expression is increased in animal models of hypertension and diabetic nephropathy, suggesting that GLUT12 would contribute to additional glucose reabsorption in the late nephron, when the glucose transport capacity in the proximal tubule is saturated due to an overload of filtered glucose [39]. These data are supported by the evidence that in the distal tubular epithelial kidney cell line MDCK, high extracellular glucose induces GLUT12 translocation to the apical membrane from the perinuclear localization, in a process mediated by mTOR signaling [78].

Expression of GLUT12 has also been found in the cytoplasm of mammary epithelial cells of pregnant rats, from where it translocates to the apical membrane during lactation. Since GLUT1, the other glucose transporter in mammary glands, is not found in the membrane in lactation, the authors suggest that GLUT12 may be the main transporter in charge of transporting glucose into the milk [40].

Regarding GLUT12 in the brain, our group has demonstrated its expression in different brain areas in mice [23]. Furthermore, we have reported an increase of GLUT12 expression in the brain of mice in models of Alzheimer’s disease (AD) where Glut1 and Glut3 (the main glucose transporters in the brain) are decreased. Indeed, β-amyloid deposition directly induces GLUT12 upregulation [23]. In line with these results, we demonstrated for the first time the increase of GLUT12 in the frontal cortex of aged subjects, that is even higher in AD aged patients [55]. Knowing that a progressive impairment of the brain´s capacity to utilize glucose and respond to insulin occurs in AD [15], these data suggest an important role of GLUT12 in this pathology.

Interestingly, although GLUT12 is a facilitative transporter, it can also mediate the entrance of glucose into the cell in co-transport with H^+^, being able to accumulate the sugar against its concentration gradient in MDCK cells [78]. Likewise, functional studies performed in Caco‐2 cells show that sugar transport is increased by H^+^ [22]. As mentioned before, GLUT12 belongs to class III of the GLUT family, which also includes the H^+^-myoinositol cotransporter [79]. This characteristic of GLUT12 suggests that it could be implicated in glucose reabsorption in the late nephron, where the tubular fluid pH is acidic [79], and in the small intestine, which presents an acidic microenvironment next to the brush border. GLUT12 can also transport substrates different from hexoses. Thus, it has been shown that it transports vitamin C from the epithelial cells of the choroid plexus into the cerebrospinal fluid, and urate from the blood into the liver, as demonstrated in cell lines and GLUT12 knockout mice [46, 69]. GLUT12 protein is also expressed in the endocrine chromaffin cells from the adrenal medulla, the anterior pituitary lobe, the gastrin-secreting pyloric glands, and the epithelial cells of the thyroid gland follicles [45], as well as in human fetal membranes and placenta [26, 27, 64, 65]. And *GLUT12* mRNA has been found in the ciliated cells and ionocytes from the airway epithelium [4].

### GLUT12 and obesity

GLUT12 protein expression in human adipose tissue was initially described upon the identification of GLUT12 [59]. Subsequently, our group demonstrated peri‐nuclear localization of GLUT12 in mouse adipocytes in which insulin, through the PI3K/AKT pathway, induces GLUT12 translocation to the membrane that parallels glucose uptake by the transporter [21], as it has been extensively demonstrated for GLUT4 [10].

**Fig. 1:**
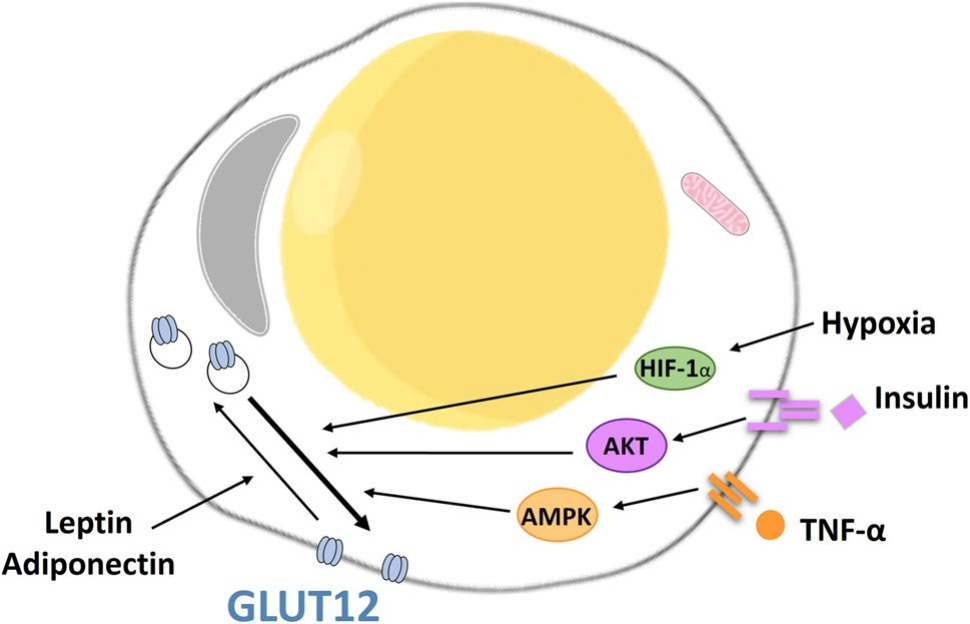
**Summary of GLUT12 regulation in adipocytes.** Insulin, TNF‐α and hypoxia induce GLUT12 translocation to the membrane from intracellular compartments, which parallels increase on glucose uptake. The adipokines leptin and adiponectin, however, reduce glucose transport by GLUT12 by triggering the transporter internalization [21]

In obesity, the accumulation of lipids in the adipose tissue triggers an inflammatory response which includes the secretion of proinflammatory cytokines such as Tumor necrosis factor-alpha (TNF‐α) [68]. In line with this data, we have reported that TNF‐α, by AMPK activation, increases glucose uptake in 3T3‐L1 murine adipocytes cell line by triggering GLUT12 translocation to the membrane [21]. The hypertrophy and hyperplasia of the adipocytes also lead to a hypoxic environment which further alters the secretion pattern of adipokines [58]. Hypoxia also induces upregulation of GLUT12 protein expression in 3T3-L1 adipocytes [21]. Accordingly, the promoter region of *GLUT12* gene (*SLC2A12*) contains hypoxia response elements [22]. The adipokines leptin and adiponectin, however, reduce glucose transport by GLUT12 by inducing the transporter internalization in 3T3-L1 adipocytes and mouse visceral adipose tissue explants. Of note, GLUT4 is upregulated by the two adipokines [21]. Figure 1 summarizes the regulation of GLUT2 trafficking in adipocytes.

In visceral adipose tissue of diet-induced obese (DIO) mice and subjects with obesity, we found that the expression of GLUT12 was decreased compared to lean controls, as it occurs for GLUT4. Moreover, the increase of Akt phosphorylation and GLUT12 expression in adipocytes induced by intraperitoneal injection of insulin in lean animals is lost in obese mice [21]. In line with these data, we have also shown that in DIO mice, where mesenteric adipose tissue contributes to intestinal inflammation through the secretion of proinflammatory cytokines [62], the amount of GLUT12 in the apical membrane of the enterocytes is lower than in lean animals [22, 24]. Furthermore, in agreement with the results found in visceral adipose tissue, intraperitoneal injection of insulin in DIO mice does not induce translocation of GLUT12 to the apical membrane of the enterocytes, compared to the increase found in control animals. These results are accompanied by *Tnf‐α* and Hypoxia inducible factor-1 *α* (*Hif‐1α*) genes upregulation in the jejunal mucosa of the obese mice [22].

GLUT12 expression is also decreased in the mesenteric adipose tissue and kidney of DIO mice, while no changes on GLUT12 amount are found in skeletal muscle [24]. In an equine model of insulin resistance, other authors have found a decrease of GLUT4 expression in the membrane of omental adipose tissue and skeletal muscle, while GLUT12 expression was unchanged [74, 75].

**Fig. 2:**
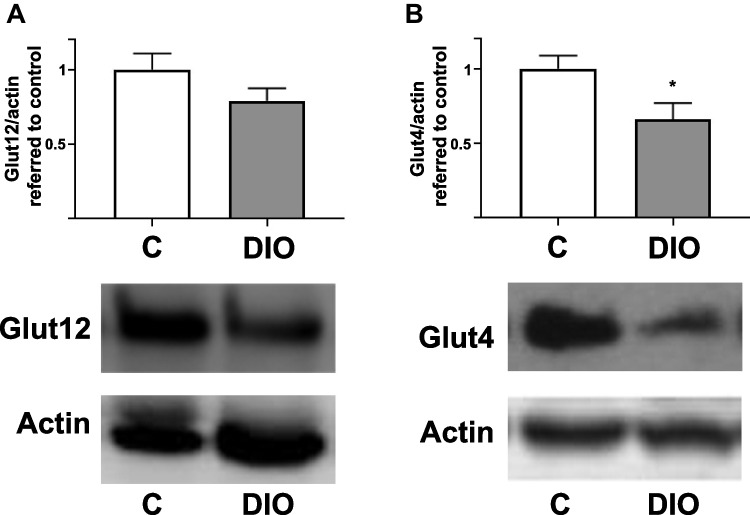
**Expression of GLUT12 and GLUT4 in interscapular brown adipose tissue (BAT) of lean (control) and diet-induced obese mice (DIO).** BAT samples correspond to the study of Gil-Itube et al. [24]. Mice were 18 months old and had been fed with DIO diet for 16 months. Upper panels, protein expression is represented as the optical density ratio between (A) GLUT12 / (B) GLUT4 and β-Actin, and expressed in fold change relative to the control group as mean ± SEM (n = 9). Bottom panels, representative Western blot images. C, Control; DIO, diet-induced obese mice. *p < 0.05 *vs*. control. Western blots were performed as previously described [24]

Brown adipose tissue (BAT) represents only 1–2% of body fat in humans [36]. Functionally, it is characterized by its thermogenic capacity of dissipating energy as heat [36] having, therefore, an important role in the regulation of energy homeostasis and the prevention of obesity [77]. BAT activity is also involved in the regulation of glucose homeostasis [47]. We have not observed significant change on GLUT12 expression in BAT from DIO mice (Fig. 2A). In agreement with the literature [43], a significant decrease of GLUT4 was found in the same animals (Fig. 2B).

As obesity, aging is characterized by a chronic inflammatory condition, frequently accompanied by the accumulation of visceral fat that eventually may lead to obesity [18, 19]. Contrary to the decrease of GLUT12 in obesity, we have found an increase of GLUT12 expression in murine small intestine, mesenteric adipose tissue, kidney [24], and brain [23] of aged mice, and in the frontal cortex of aged individuals [55]. These data suggest that in obesity, the excessive energy condition would induce GLUT12 downregulation in some organs whereas in aging, the decrease of metabolism would be a stimulus to upregulate GLUT12 [24].

As GLUT4 knockout mice is surprisingly normoglycemic [37], and the skeletal muscle retains the capacity of responding to insulin [66], GLUT12 has been proposed as an important transporter of glucose when GLUT4 is impaired in the muscle [67]. Accordingly, GLUT12 overexpression in mice improves whole body insulin sensitivity [57]. We can argue that GLUT12 could be a glucose transporter of clinical interest in conditions where insulin resistance is present. Interestingly, Fam et al. [17] reported no change of GLUT12 and GLUT1 total expression in their model of GLUT4 knockout mice, hypothesizing that other GLUTs may contribute to regulate the glucose homeostasis. It would be of interest to study whether the relative amount of GLUT12 in the plasma membrane is increased in this model, explaining the maintenance of normoglycemia.

### GLUT12 and cancer

GLUT12 protein is overexpressed in human breast tumors compared to non-tumoral breast tissue [60]. In MCF-7 cells, metabolites that drive to oncogenic proliferation such as estradiol and epidermal growth factor increases GLUT12 protein levels [41]. Interestingly, upregulation of GLUT12 has also been found in MCF-7 cells incubated with a high glucose concentration; under this condition, the increase of cell migration observed is abolished after GLUT12 inhibition [44]. Most recently, it has been demonstrated the suppression of proliferation, migration and invasion of breast cancer cells and xenografted tumors, after inhibition of GLUT12-mediated Warburg effect [61].

**Fig. 3:**
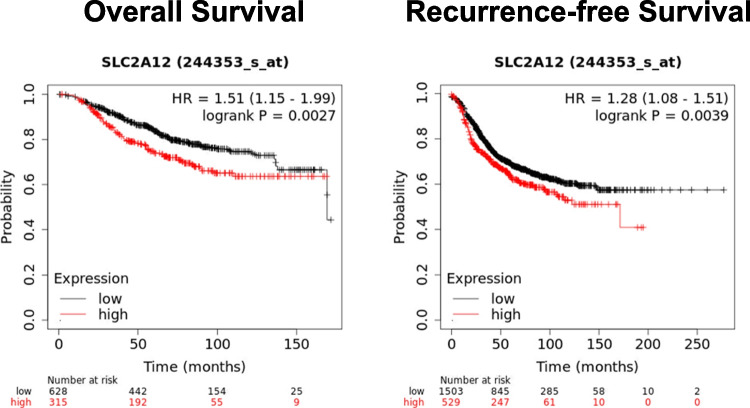
**Kaplan–Meier survival plots of SLC2A12 expression level in breast cancer patients.** KM Plotter Online Tool (http://www.kmplot.com) was used as a clinical outcome prediction tool. The parameters evaluated were Overall Survival (Left panel) and Recurrence-Free Survival (Right panel). Patients were distributed according to the best cutoff values of the gene expression (lowest p value) into “high” *vs.* “low”

When we analyzed *GLUT12* mRNA expression levels in breast cancer patient cohorts using the KMplotter online tool, we observed a decrease in overall survival and recurrence-free survival in patients with high *GLUT12* expression (Fig. 3).

In addition, immunohistochemistry studies revealed higher expression of GLUT12 in a cohort of triple negative breast cancer (TNBC) patients compared to non-TNBC. Within TNBC patients, those showing higher GLUT12 expression presented shorter overall survival and recurrence-free survival [61].

**Fig. 4:**
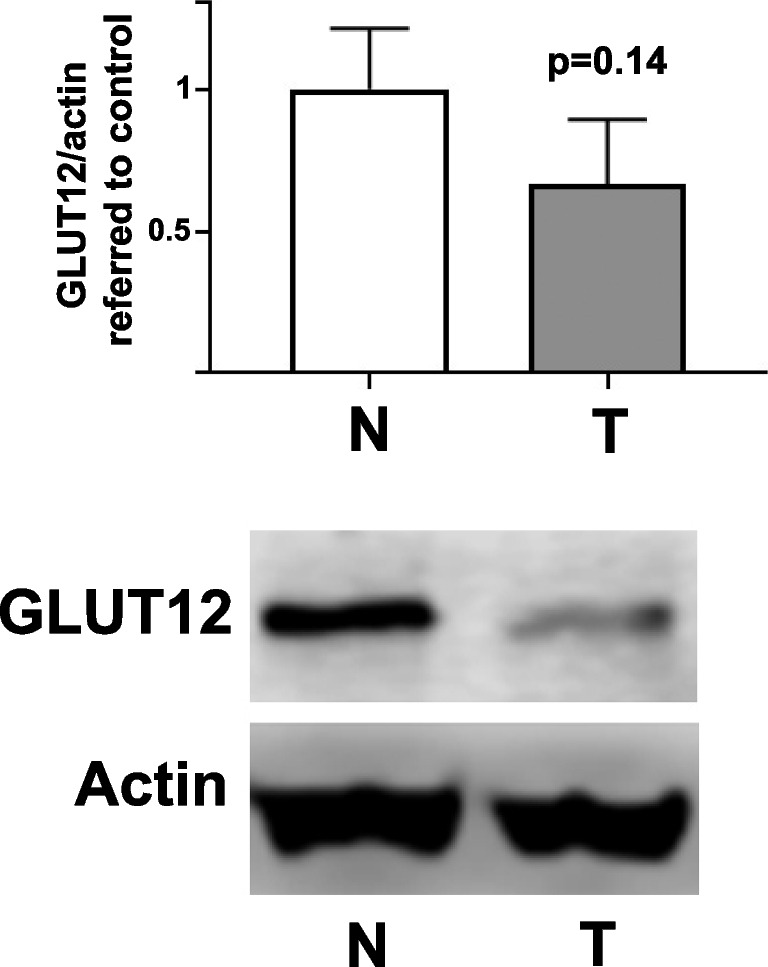
**Expression of GLUT12 in lung adenocarcinoma.** Samples were provided by the Biobank of the University of Navarra and processed following standard operating procedures, approved by the Ethics Committee of the University of Navarra (n. 2011.006mod1). Upper panel, protein expression is represented as the optical density ratio between GLUT12 and β-Actin and expressed in fold change, relative to the control group, as mean ± SEM (n = 6). Bottom panel, a representative Western blot image. N, Non-tumor (control); T, tumor. *p < 0.05 *vs*. control. Western blots were performed as previously described [24]

Differently, high levels of *GLUT12* mRNA have been significantly associated with better overall survival in lung adenocarcinoma patients [16]. And, at the protein level, we have found in lung adenocarcinoma tumors a decreased trend on GLUT12 expression, compared to non-tumoral adjacent tissue (Fig. 4).

GLUT12 and GLUT1 are expressed in the plasma membrane and cytoplasm of prostate carcinoma cell lines. Interestingly, while GLUT12 is absent in normal prostate tissue and present in primary prostate carcinomas, an inverse pattern has been observed for GLUT1 [13]. Remarkably, in prostate cell lines the inhibition of GLUT12 halted the cell growth [76].

Regarding the digestive tract, *GLUT12* has been detected in oral squamous carcinoma cell lines [49]. Most important, Cao et al. [12] have recently demonstrated in gastric cancer cell lines, that GLUT12 expression induces proliferation switching the oxidative phosphorylation pathway to a highly glycolytic metabolism. The same authors show that GLUT12 is upregulated, in an androgen receptor-manner, to counteract the treatment with everolimus, a mTOR kinase inhibitor which triggers apoptosis. Accordingly, inhibition of the androgen receptor abolishes the increase of GLUT12 expression by everolimus, both in gastric cancer cells and a xenografted mice model, favoring the antitumoral effects of the drug, as occurs by knocking down GLUT12 [12].

The PI3K-AKT-mTOR pathway comprises a node signaling for growth, proliferation, and survival in the tumor cells. The AKT upregulation of GLUT1 in tumor cells was initially described in the late 90 s of the past century [6], being a mechanism for an increase of glucose uptake. In this line, GLUT12 translocates to the membrane after PI3K/AKT activation by insulin in human muscle [45] and mouse adipocytes [21]. Moreover, mTOR inhibition blocks GLUT12 trafficking to the membrane under high glucose conditions in renal cells [78]. Further research is needed to clarify the reprogramming of GLUT12 function in cancer cells and its regulation by PI3K signaling pathway.

**Fig. 5:**
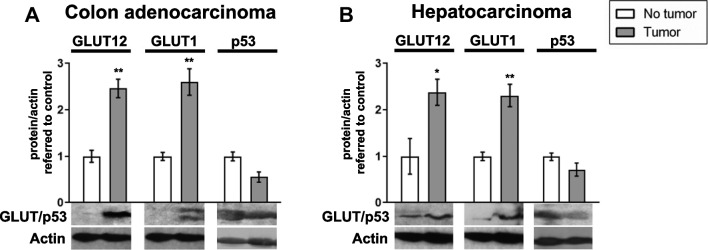
**GLUT12 and GLUT1 expression in colon adenocarcinoma and hepatocellular carcinoma.** Samples were provided by the Biobank of the University of Navarra and processed following standard operating procedures, approved by the Ethics Committee of the University of Navarra (n. 2011.006mod1). A) GLUT12, GLUT1 and p53 expression in colon adenocarcinoma biopsies and healthy adjacent tissue. B) GLUT12, GLUT1, and p53 expression in hepatocellular carcinoma and healthy adjacent tissue. Protein expression is represented as the optical density ratio between GLUT12, GLUT1 or p53 protein and β-Actin and expressed in fold change, relative to the healthy tissue, as mean ± SEM (n = 5). Representative western blots are shown. *p < 0.05, **p < 0.01 *vs*. healthy colon/liver. Western blots were performed as previously described [24]

In patients suffering from colon adenocarcinoma and hepatocarcinoma, we have found an increase on GLUT12 and GLUT1 protein expression in tumors compared to adjacent non-tumor tissue, while the tumor suppressor p53 showed a diminishing trend (Fig. 5A and B). To our knowledge, this is the first time that expression of GLUT12 in liver/hepatocarcinoma is reported. Apoptosis depends on glycolytic rates [72], and the tumor suppressor p53, which influences both the apoptosis [80] and the balance between glycolysis and oxidative phosphorylation [8], binds directly to the promoters of GLUT12 and GLUT1 repressing their expression in cell lines from different types of cancer [81]. Further, p53 expression is low or mutated in MCF-7, A549, HT-29 and RH-36 cell lines [11, 48], where GLUT12 is expressed [56], while is high in neuroblastoma cell lines, in which GLUT12 is not detected [48, 56, 70].

Hypoxia is one of the common features of cancer. The reduction of oxygen availability induces HIF-1, a key regulator of cancer cell proliferation [34], which contributes to the anaerobic glycolysis through the stimulation of a number of genes that mediate glycolysis and angiogenesis [14], including GLUT1 and GLUT3 [7, 63]. As mentioned earlier, hypoxia increases GLUT12 expression in adipocytes [21]. In line with our previous data, we have also shown an increase on GLUT12 amount in the hypoxic center of spheroid cultures of the colorectal adenocarcinoma HT29 cell line, in comparison with the low expression in the cell monolayers [56]. This result would indicate an induction of the activity of the transporter under hypoxic conditions, that would drive to an increase of glycolysis; which, in turn, would be important to start the tumor cell response to hypoxic conditions before the angiogenesis is initiated.

**Fig. 6:**
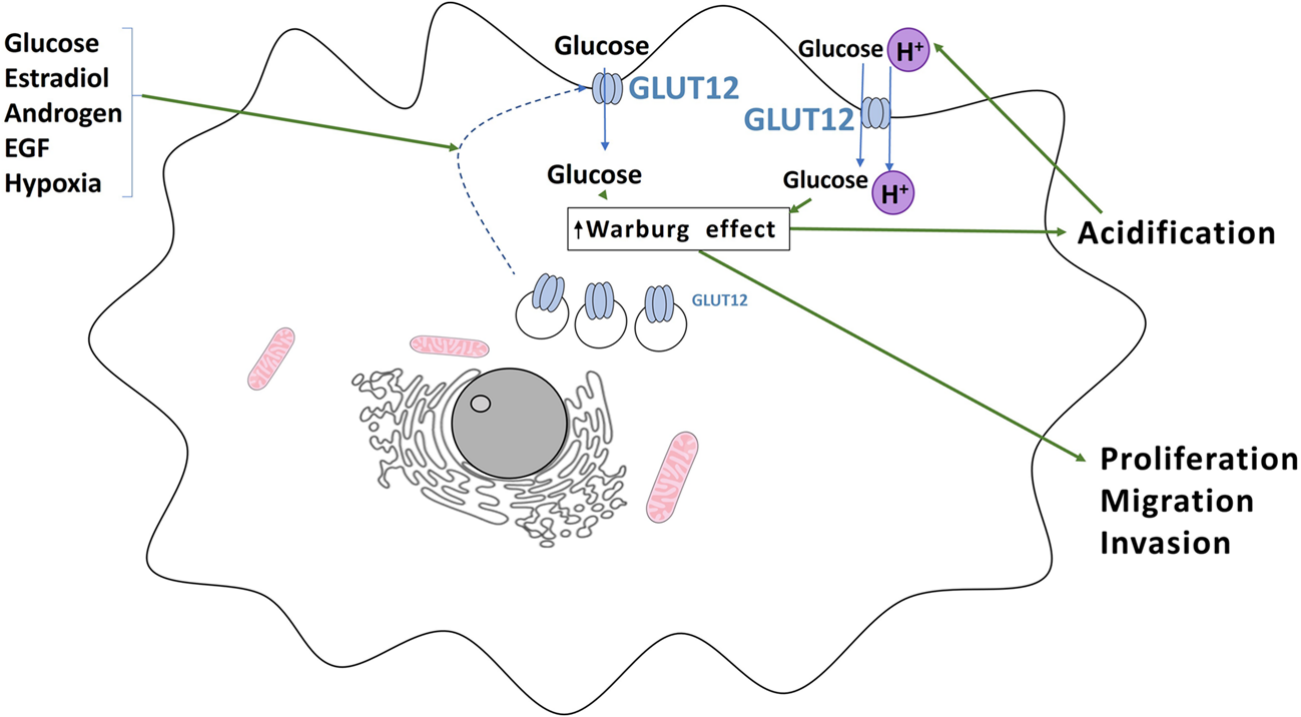
**Summary of GLUT12 regulation in tumor cells.** In tumor cells, increase of glucose uptake by GLUT12 is stimulated by glucose and the hormones estradiol, epidermal growth factor (EGF) and androgens, which induce trafficking of the transporter to the plasma membrane. In this way, GLUT12 provides the tumor cell with the glucose it needs to produce the required ATP through the aerobic glycolysis (“Warburg effect”), for proliferation, migration and invasion. As a consequence of the Warburg effect, extracellular acidification also provides GLUT12 with the electrochemical gradient of H^+^ to cotransport and accumulate glucose inside the cell, further feeding the aerobic glycolysis. Hypoxia, one of the common features of cancer, also upregulates GLUT12, thus rising glucose uptake, which increase anaerobic glycolysis before angiogenesis is initiated to meet the oxygen demands of the tumor [41, 44, 56, 61, 76, 79]

On the other hand, in cancer, the increase on glycolytic metabolism and lactate production due to the Warburg effect leads to an acidic environment [33]. Since GLUT12 can act as a H^+^/glucose symporter [79], we hypothesize that this mechanism will allow GLUT12 to accumulate the sugar inside the tumor cell, thus contributing to its proliferation. Figure 6 summarizes GLUT12 regulation in tumor cells.

In addition to the involvement of GLUT12 in the proliferation of cancer cells, GLUT12 might be directly involved in cancer cell evasion and metastasis. Accordingly, in the cell line HEK293T cells, activation of the transcription factor Twist-related protein 1 (TWIST1), a key protein for epithelial to mesenchymal transition, increases *GLUT12* along with *GLUT1* and *GLUT3* mRNA in an insulin-independent manner by directly binding to their promoters [51].

Table 1 summarizes the signaling proteins involved in oncogenic processes and obesity that regulate GLUT12 expression and trafficking to the membrane.

**Table 1:** Signaling proteins involved in oncogenic processes and obesity that regulate GLUT12 expression and trafficking to the membrane

Protein	Cell/Tissue	Reference
PI3K/AKT/mTOR	3T3-L1 murine adipocytes	21
	Human skeletal muscle biopsies	67
	Madin-Darby canine kidney cell line MDCK	78
AMPK	3T3-L1 murine adipocytes	21
	Colon adenocarcinoma cell line Caco-2	22
CaMLL2-AMPK	Human prostate cancer cell line LNCaP	76
Estrogen	Breast cancer cell line MCF-7	41
Androgen	Human prostate cancer cell line LNCaP	76
	Gastric cancer cell lines SGC-7901, HGC-27/Xenografted mice models	12
EGF	Breast cancer cell line MCF-7	41
Twist1	HEK293T cells	51

## Conclusions and future perspectives

GLUT12 is one of the less investigated members of the SLC2 family, which displays unique functional properties, but whose physiological role in the organism still needs to be elucidated. Here, we have collected all the information in the literature about GLUT12 in obesity and cancer. It would be interesting to elucidate whether in cancer patients with obesity, in fact, the decrease of GLUT12 in adipocytes inversely correlates with the increase of GLUT12 in the tumor, and whether this correlation could contribute to the worse prognosis of these patients with obesity.

## Data Availability

The data that support the findings of this study are not openly available due to reasons of sensitivity and are available from the corresponding author upon reasonable request.
